# Challenges in Echocardiography for the Diagnosis and Prognosis of Non-Ischemic Hypertensive Heart Disease

**DOI:** 10.3390/jcm13092708

**Published:** 2024-05-04

**Authors:** Nikolaos P. E. Kadoglou, Angeliki Mouzarou, Nikoleta Hadjigeorgiou, Ioannis Korakianitis, Michael M. Myrianthefs

**Affiliations:** 1 Medical School, University of Cyprus, 215/6 Old Road Lefkosias-Lemesou, Aglatzia, Nicosia CY 2029, Cyprus; 2 Department of Cardiology, Pafos General Hospital, Paphos CY 8026, Cyprus; 3 Department of Cardiology, Nicosia General Hospital, Nicosia CY 2029, Cyprus

**Keywords:** hypertensive heart disease, echocardiography, prognosis, speckle tracking

## Abstract

It has been well established that arterial hypertension is considered as a predominant risk factor for the development of cardiovascular diseases. Despite the link between arterial hypertension and cardiovascular diseases, arterial hypertension may directly affect cardiac function, leading to heart failure, mostly with preserved ejection fraction (HFpEF). There are echocardiographic findings indicating hypertensive heart disease (HHD), defined as altered cardiac morphology (left ventricular concentric hypertrophy, left atrium dilatation) and function (systolic or diastolic dysfunction) in patients with persistent arterial hypertension irrespective of the cardiac pathologies to which it contributes, such as coronary artery disease and kidney function impairment. In addition to the classical echocardiographic parameters, novel indices, like speckle tracking of the left ventricle and left atrium, 3D volume evaluation, and myocardial work in echocardiography, may provide more accurate and reproducible diagnostic and prognostic data in patients with arterial hypertension. However, their use is still underappreciated. Early detection of and prompt therapy for HHD will greatly improve the prognosis. Hence, in the present review, we shed light on the role of echocardiography in the contemporary diagnostic and prognostic approaches to HHD.

## 1. Introduction

Arterial hypertension (AH) is a very common disease worldwide and a strong risk factor for cardiovascular diseases or conditions, including atrial fibrillation (AF), perioperative ischemia, coronary artery disease (CAD), cardiac-related rehospitalization, valvular diseases, and acute aortic syndromes (aortic dissection, intramural hematoma, or aortic ulcer) [1]. Although the cutoff criteria for AH vary between the American and European guidelines, there is substantial similarity in most recommendations for the management of AH and its complications [2,3,4]. Among AH-derived complications, hypertensive heart disease (HHD) has been widely recognized and refers to disturbed cardiac structure and function, affecting the left ventricle (LV), left atrium (LA), and coronary arteries as a result of prolonged exposition to high blood pressure (BP) [5]. Nevertheless, there is no universal consensus on the definition of HHD. Therefore, the diagnosis of HHD is based on clinical history, imaging modalities like echocardiography, and functional or anatomical tests for myocardial ischemia, to identify all the possible changes in the myocardium and coronary arteries.

From the pathophysiological perspective, unmanaged high BP induces high afterload and high LV filling pressures, leading to structural changes such as left ventricular hypertrophy (LVH) and fibrosis, and LA enlargement [6]. Besides this, HHD relates to systolic and diastolic LV dysfunction or a combination of them. AH stands out as a primary risk factor for heart failure (HF) development. Recent evidence suggests the co-existence of AH in 76% of newly diagnosed HF cases [7], and individuals with AH have an almost twofold higher lifetime risk of HF development compared to those with normal BP [8]. AH stands out as the predominant and highly impactful morbidity factor in heart failure with preserved ejection fraction (HFpEF), with a prevalence of 80% in the Get with the Guidelines (GWTG) initiative. The most formidable challenge in the diagnosis and management of HfpEF lies within its high incidence, affecting a high percentage of individuals [9,10]. Moreover, numerous epidemiological studies have unveiled the link between AH and CAD, a significant HF risk factor^3.^ Individuals with HHD face an elevated risk of developing CAD affecting both the large and small arteries (microvascular disease). Notably, the INTERHEART study demonstrated that 25% of the population-attributable risk for myocardial infarction can be ascribed to AH [11].

Despite the interplay between AH and CAD, one of the most frequent challenges in clinical practice is to diagnose HHD in patients without underlying CAD. Echocardiography is an easily performed, cheap, repeatable, and immediately available modality to estimate the cardiac structure and function [12]. During the working-up diagnosis of HHD, several classic echocardiographic indices (e.g., LVH) have been proposed. Besides this, novel echocardiographic parameters (e.g., speckle tracking) may be useful to identify cardiac changes due to HHD at an early stage. It is also worth noting that the search for markers of subclinical dysfunction of the cardiovascular system is gaining increasing interest, and random measurement of biomarkers like cardiac troponin may be useful in this regard [13]. Prompt therapy may inhibit cardiac remodeling and attenuate cardiovascular risk [14]. Most importantly, imaging modalities have been proposed for individuals’ risk stratification in the context of precision medicine [15]. Thereby, the echocardiographic indices may not only help with diagnosis, but they may also give an impression of patients’ prognosis or their response to therapy. High-risk patients should receive more intensive therapy compared to low-risk patients [16]. Therefore, there are multiple benefits from echocardiography’s application in HHD.

The purpose of this paper is to provide an overview of the classic and novel echocardiographic findings and their role in the diagnosis and prognosis of HHD without CAD. An objective knowledge of the potentiality and challenges of this imaging modality might promptly detect HHD, prevent its progression to HF, or even reverse HF.

## 2. Pathophysiology

Chronic AH triggers pathological processes, leading to structural and functional disturbances in the myocardium, like hypertrophy, fibrosis, and ischemia [6,17]. Chronic or sustained AH leads to pressure overload of the left cardiac cavities, induces change in gene expression, and confers simultaneous changes in the extracellular matrix and myocytes hypertrophy. Structurally, the LV walls become thicker, with or without a simultaneous increase in absolute myocardial mass, as a compensatory mechanism to pressure overload. Depending on whether there is LV dilation, four patterns of hypertrophy are distinguished: eccentric non-dilated, eccentric dilated, concentric non-dilated, and concentric dilated [18]. This is a classification from the pathophysiology and anatomy perspective, which differs from that based on the echocardiography findings mentioned in the next section. Non-hemodynamic factors, such as the renin–angiotensin–aldosterone system, demographic factors (gender and ethnicity), obesity, and genetics, play a significant role in myocardial thickening. It is worth noting that it is not fully understood why patients with HHD develop a specific LVH pattern.

Another integral element of HHD is myocardial fibrosis [19], either interstitial (reactive, diffuse) or replacement (reparative). Myocardial reactive fibrosis involves the accumulation of fibrous connective tissue in the interstitial and perivascular spaces, without a significant loss of myocardial cells. Replacement fibrosis, on the other hand, consists of scar tissue formed due to the loss of myocardial cells and their replacement by connective tissue following myocardial infarction or myocardial death from other causes [20]. It is still unclear whether those two types of myocardial fibrosis represent distinct processes, as they may occur simultaneously^6^. Hemodynamic factors, such as chronic pressure overload causing myocardial stress or injury, lead to increased collagen production (type I, type III) in the myocardium as a reparative mechanism. Non-hemodynamic factors, such as the renin–angiotensin–aldosterone system, also play a significant role in myocardial fibrosis^6^.

HHD eventually may progress towards HF, where the initial myocardial hypertrophy and fibrosis result in myocardium stiffening and systolic and diastolic LV dysfunction. Eccentric hypertrophy is more likely to lead to HF with reduced ejection fraction (HFrEF), while concentric hypertrophy may lead to HF with preserved ejection fraction (HFpEF). Additionally, changes in the LA morphology begin in the early stages of HHD despite the normal-sized LV^18^. In particular, microstructural changes (LA wall changes) precede macroscopic alterations (LA dilatation), resulting in a functionally impaired atrium despite its normal size. LA remodeling in HHD is once again the result of both hemodynamic factors (increased afterload) and non-hemodynamic factors (neurohormonal activation) [21]. The increased LV afterload leads to elevated filling pressures within the LA, consequently increasing its wall tension. As an adaptation, the LA may reshape, affecting first its contractility. Non-hemodynamic factors, such as the activation of the renin–angiotensin–aldosterone system, the release of natriuretic peptides, and endothelin-1, also play a significant role. Those factors trigger inflammatory cell accumulation, leading to fibrosis and further remodeling of the LA. Additionally, there is an increase in sympathetic nervous system activation due to neurohormonal activation. In the context of HHD, the involvement of norepinephrine plays a pivotal role in the pathophysiological cascade. The sequence begins with increased sympathetic nerve activity, particularly in young hypertensive individuals, leading to elevated heart rate, cardiac output, renal vascular resistance, and blood pressure. Norepinephrine, along with other humoral factors like angiotensin II, contributes to a hemodynamic profile that induces adaptive changes in cardiac structure, ultimately culminating in hypertensive LVH. The significance of norepinephrine becomes apparent as its high levels or interactions with humoral factors potentiate structural changes in the heart [22].

Despite the acknowledged importance of norepinephrine in the development of LVH, there exists a notable discrepancy in the effects of antiadrenergic drugs on regression. While α1-adrenergic blockers, such as doxazosin, demonstrate favorable effects on reversing LVH, the clinical consequences may be not always beneficial with reducing LA mass, as evidenced by an increased risk of HF incidence during treatment [23].

AF is frequently observed not only in patients with a dilated LA, but also when atrial fibrosis is present. Speckle tracking and cardiac magnetic resonance (CMR) have clarified this point [24]. It is worth mentioning that several mechanisms have been proposed to explain how systemic AH leads to remodeling of the right ventricle (RV), and fewer have been proposed for the right atrium (RA). However, the whole process is not yet fully understood. The same pathophysiological steps present in the left cavities are involved in the right cavities as well: hypertrophy of myocardial cells, fibrosis, and alterations in the composition of the extracellular matrix. The RV myocardium becomes thicker and dilated, accompanied by RA dysfunction^22^. Finally, persistent AH leads to arteriosclerosis of the vessels in circulation, as well as microvascular rarefaction. The co-existence of hypertrophic myocardium and arterial stiffness increases the LV afterload and precipitates LV remodeling, affecting the overall systolic and diastolic function of the heart.

## 3. Classical Echocardiographic Indices

Echocardiography plays a pivotal role in identifying structural changes in individuals with AH, leading to the development of HHD. Table 1 summarizes the echocardiographic parameters, the challenges and their prognostic value.

### 3.1. Left Ventricular Hypertrophy

LVH is a common manifestation of HDD and defined as LV thickening (≥12 mm) or increased LV mass (LVM) [25]. LVH has long been used as a risk factor in individuals without known cardiovascular disease. Hence, its detection stratifies the cardiovascular risk of patients with and without HHD and has considerable impact on patients’ management and prognosis. Both LV thickening and increased LV mass rely on linear measurements of LV dimensions on 2-dimensional (2D) images, while LV mass calculation uses geometric assumptions regarding the LV shape [26]. The echocardiography image quality is of paramount importance for measurements. Moreover, there is a high intra-observer and interobserver variability, even with high image resolution 2D echocardiography. The presence of a moderator band, different sites for dimension measurements in the parasternal long-axis (PLAX) and parasternal short-axis (PSAX) views, and the miscalculation of wall thickness at the papillary muscles’ origin may attenuate the reliability of measurements. Thus, the 2D echocardiographic assessment of LV mass has significant drawbacks and tends to overestimate LV mass in comparison to CMR imaging [27]. This is the case when image quality is suboptimal. The recent advances in 3D echocardiography may compensate for the abovementioned limitations, with less overestimation of LV mass than in CMR [28]. However, an optimal 2D image quality is a prerequisite for valid 3D echocardiography, while it is difficult to detect changes in LV mass over time. Therefore, a technically perfect echocardiographic examination from an experienced operator is deemed necessary. Another echocardiographic index of LVH is the calculated relative wall thickness (RWT) based on the formula 2PWT/LVDd (PWT, posterior wall thickness; LVDd, left ventricular diastolic diameter).

**Figure 1:**
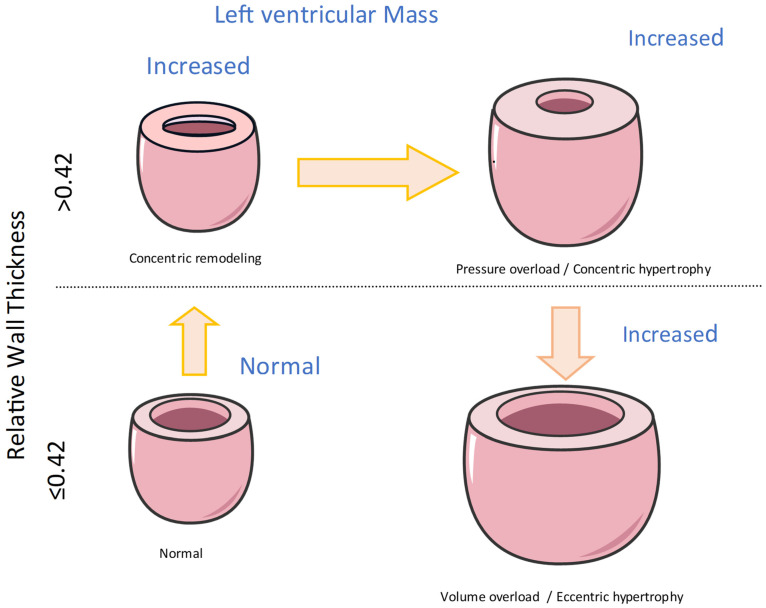
Common patterns of left ventricular hypertrophy in hypertensive heart disease.

Three main patterns of LVH based on echocardiography, which differ from the pathophysiological classification, have been first described: (a) concentric hypertrophy, (b) eccentric hypertrophy, and (c) concentric remodeling^6^. This classification is commonly used in echocardiography labs. The first two, concentric and eccentric LVH, are the two main patterns of LVH. That classification is based on the following three parameters with their cut-off values: (1) the relative wall thickness (RWT) ≥ 0.42, (2) a left ventricular mass index (LVMI) ≥ 95 g/m^2^ in females or ≥115 g/m^2^ in males, and (3) an end-diastolic diameter (LVEDD) ≥ 5.3 cm in females or ≥5.8 cm in males in hypertensive individuals; these parameters enhance clinical understanding and guide appropriate interventions. Adding the end-diastolic volume refines the classification of LVH in four distinct patterns: eccentric non-dilated, eccentric dilated, concentric non-dilated, and concentric dilated hypertrophy (Figure 1).

Since LV mass relates to body size, indexing its values to body surface area (BSA) has been used in population-based studies for reference values. LV mass indexed to BSA underestimates the prevalence of LVH in obese patients [29]. An alternative to traditional BSA indexing is using height alone for adjusting cardiac parameter measurements, particularly relevant in HHD assessments. This method aims to provide a more nuanced evaluation by considering an individual’s height as the sole determinant, addressing variations in body size not fully captured by BSA. Height indexing enhances precision in evaluating cardiac structure and function, providing a more individualized assessment of dimensions. Utilizing height as a reference in obese patients acknowledges the significance of differentiating between overall body size and its composition. This approach enhances the ability to gain a more accurate understanding of cardiac health within this specific population [30,31]. The cutoff values, based on height alone, for the left ventricular mass index are established at equal to or greater than 45 g/m^2.7^ in females and equal to or greater than 49 g/m^2.7^ in males.

The prognostic value of LVH is unambiguous. The pattern of increased LV mass has an added value to LVH in general and to the hypertensive population [32,33]. Since the 1990s, it has been demonstrated that LVH is a strong factor for cardiovascular mortality [34]. Ten-year follow-ups of hypertensive patients have outlined the prognostic power of LVH and ventricular arrhythmias [35]. An indexed LV mass has better prognostic ability and may be preferred instead of absolute values of LVH. However, the question of whether to use BSA or height for LV mass indexing is the subject of debate. Another proof of the prognostic impact of LVH derives from studies using anti-hypertensive medications. Targeted LVH regression has been accompanied by cardiovascular benefits [36].

### 3.2. Left Ventricular Systolic Function

The conventional assessment of left ventricular systolic function relies on metrics like the LV ejection fraction (LVEF) and endocardial fractional shortening (FS). However, their application, primarily at the endocardial surface, sparks concern about their relevance in the context of LVH. It becomes apparent that these measurements may tend to overstate LV systolic function [37,38].

An increasingly embraced metric is stress-corrected midwall fractional shortening. This can provide a more nuanced reflection of systolic function, particularly beneficial in cases of LVH. Unlike left ventricular endocardial FS, this parameter paints a more accurate picture [39]. The LV systolic function assessment may face challenges, especially in patients with inadequate quality of acoustic windows, like obese individuals or those with chronic obstructive pulmonary disease. Unfortunately, being overweight or obese are common predisposition factors of HHD, preventing early echocardiographic diagnosis. Moreover, regional wall motion abnormalities (WMAs) may blur the systolic function assessment. Simpson’s biplane technique is the most reliable, but it is still based on geometrical assumptions and faces important limitations. [40]. All echocardiographic methods rely on good acoustic windows for clear delineation of the blood/endocardial border, vital for accurate measurement/tracing. The alternative administration of contrast agents has proven effective in improving LVEF determination in patients with challenging acoustic windows [41].

Additionally, in terms of accuracy, studies have consistently shown that echocardiography tends to underestimate the LVEF when compared to the more advanced imaging capabilities of CMR [42]. A reduced LVEF, or even a low normal LVEF, has been long associated with poor prognosis in HHD, which underscores the necessity for accurate measurements and intensive therapy. In the context of HFrEF, the prognosis is reduced proportionally to the degree of LV systolic impairment [43].

### 3.3. Left Atrium Dilatation

In the context of HHD, LA enlargement signifies an early and prevalent structural change. Interestingly, the specific geometric pattern of LVH does not affect the size of the LA in the initial stages of essential AH, whether expressed in terms of diameter or volume. What emerges as a critical determinant of LA enlargement, independent of potential confounding factors, is the left ventricular mass index (LVMI) [44]. The greatest anterior–posterior dimension at the level of the aortic root of the LA has long been discussed as an unambiguous index of LA enlargement and it is highly dependent on the perpendicularity of the axis. Suboptimal views, angled views, or bad visualization of the posterior wall may lead to miscalculation of the LA dimensions.

To quantify LA volume, methods such as area–length or modified Simpson’s are employed, typically normalized for BSA and presented as the left atrial volume index (LAVI) in ml/m^2^. The normal range of the LAVI extends up to 34 mL/m^2^ [45]. Ensuring the avoidance of foreshortening in the long axis of the LA is critical for maintaining precision in the evaluation process [46,47].

Comparative studies between transthoracic echocardiography (TTE) and CMR have consistently demonstrated the underestimation of both LA and RA volumes in TTE. Discrepancies between imaging modalities may stem from poor endocardial definition and foreshortening in echocardiography, particularly evident in standard apical views, impacting the lateral atrial walls [48].

Notably, the LAVI has been related to mortality risk, regardless of the left ventricular geometry, in a substantial cohort of patients exhibiting preserved LV systolic function [49,50]. LA enlargement has been considered as a bad prognostic factor in patients with HHD and HFrEF [51]. On the other hand, it may be a consequence of HFpEF, which interplays with AF occurrence and other co-morbidities [52]. Recent studies indicate that measuring minimal left atrial volume (LAVmin) at LV end-diastole, when the left atrium is directly exposed to LV end-diastolic pressure, may offer a closer correlation to LV filling pressure and clinical outcomes compared to maximal left atrial volume (LAVmax) [53,54].

This suggests that LAVmin could be a more effective marker for LA structural remodeling. It is noteworthy that, while the prognostic value of LAVmax in patients with HFpEF is contentious, there are limited data on the prognostic value of LAVmin in HfpEF patients, necessitating further research [55,56,57,58]. The adaptation to BSA leads to the calculation of the LAVmin index (LAVImin), whose power to predict HF hospitalization appears to be notably stronger for individuals without a history of AF compared to those with AF, especially when juxtaposed with LAVImax^58^.

### 3.4. Diastolic Dysfunction

Mitral inflow measurements play a crucial role in assessing diastolic function. Parameters such as E and A velocity, their ratio (E/A), the deceleration time of E velocity, and the isovolumic relaxation time provide valuable insights. Notably, in hypertensive individuals, a normal in-treatment transmitral flow pattern serves as an indicator of a lower risk for HF, irrespective of BP levels. Although the implementation of antihypertensive therapy in patients with LVH improves mitral inflow patterns, this has not been correlated with a reduction in cardiovascular morbidity and mortality [59]. On the other hand, a high E/A ratio (>2) has been associated with grade III diastolic dysfunction, indicating high LV filling pressures and poor prognosis, especially when HF has been already developed [60,61].

The main limitation of echocardiography in diastolic dysfunction assessment is the clustering of numerous parameters and inaccuracy in blood flow calculation. The proper alignment of the ultrasound beam is pivotal; color flow echocardiography may guide the alignment of sample volume parallel to flow [62]. Using the lowest filter setting is advised to capture the full velocity profile [63].

The pulsed tissue Doppler-derived E′ velocity of the mitral annulus is an essential component in the evaluation of cardiac diastolic function. A decline in the septal (<7 cm/s) and lateral e′ (<10 cm/s) and an increased average ratio (E/e′ ≥14) signal compromised LV relaxation. It is worth emphasizing the predictive capability of the E/E′ ratio for primary cardiac events in a hypertensive population without established cardiac disease [64]. The additional assessment of late (atrial) diastolic velocity (A′), may be influenced by LA function and LV end-diastolic pressure, but its diagnostic and prognostic values have not been established.

**Figure 2:**
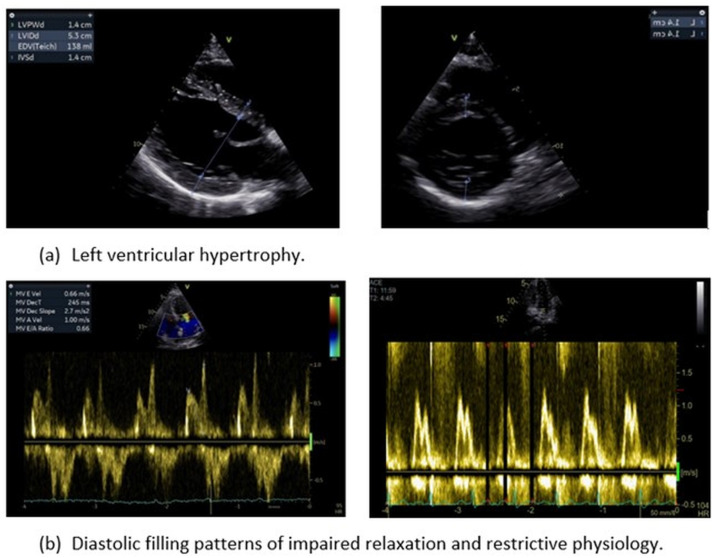
Classical echocardiographic indices of hypertensive heart disease.

The evaluation of pulmonary venous flow pattern, when obtainable, has been implicated as an independent diagnostic criterion and a valid predictor of cardiovascular events in essential AH. A high S/D ratio per se is independently associated with an increased cardiovascular disease risk in hypertensive patients [65]. Furthermore, the concomitant high pulmonary venous systolic-to-diastolic wave ratio (S/D, normal values: male < 1.51, female < 1.66) and a low E/A ratio exert predictive value in HHD^65^. In TTE, the S,D flow data are usually acquired from the orifice of the right upper pulmonary vein in the apical four-chamber view. Color flow Doppler should be employed to accurately place the sample volume 1 to 2 cm into the pulmonary vein, acknowledging that the far-field nature of the structure may limit the quality of the recording [66]. Overall, the echocardiographic assessment of diastolic function has several limitations, including dependence on the number of Doppler measurements, potential interobserver variability, and acoustic window challenges in the hypertensive population. In contrast, CMR has the advantages of precise volumetric measurements and tissue velocity mapping, independent of acoustic window quality [67] (Figure 2).

### 3.5. RV Systolic Function

RV systolic dysfunction is not only a recognized marker of adverse prognosis in HF but is also linked to poor prognosis in HHD [68]. All the following parameters have been associated with negative prognosis in the whole spectrum of cardiac diseases. On the other hand, RV measurements are usually influenced by volume load or are based on geometrical assumptions that attenuate the accuracy of RV systolic function estimation.

Echocardiographic evaluation of the RV systolic function in HHD involves a comprehensive assessment of the following various parameters of the right cavities: tricuspid annular plane systolic excursion (TAPSE), systolic velocity of the tissue Doppler of the tricuspid valve (TV) annulus, right ventricular ejection fraction (RVEF), Fractional Area Change (FAC), pulmonary artery systolic pressure (PASP), size of RV, and right ventricular systolic pressure (RVSP). A comprehensive echocardiographic assessment and early detection of right cavity dysfunction may contribute to risk stratification and tailored clinical management of individuals with HHD [69].

The TAPSE method stands out for its simplicity and reproducibility, but it is susceptible to variations in load and angle. This technique involves measuring edge-to-edge the excursion of the tricuspid annulus during systole with a swift sweep speed of ≤100 mm/s. In cases of severe tricuspid regurgitation (TR), TAPSE may exhibit pseudo-normalization due to volume overloading [70]. In parallel, the tissue Doppler RV systolic wave velocity may assist in assessing the longitudinal function of the basal RV free wall, lacking a comprehensive evaluation of global RV function. Similar to TAPSE, it is also angle- and load-dependent, requiring precise alignment of the ultrasound beam with the lateral TV annulus, essentially capturing only the longitudinal function of the RV base [71].

The assessment of RV size and systolic function through conventional echocardiography should be conducted in all patients with HHD, considering RV loading conditions. It is crucial to employ a multi-parametric approach and utilize various echocardiographic views to ensure accurate evaluation, especially when there is a discrepancy between different echocardiographic parameters [72]. This comprehensive approach enhances the precision and reliability of the interpretation of findings related to RV size and function.

HFpEF is acknowledged as a contributor to pulmonary hypertension (PH) [73]. The estimation of PH relies on the detection of the TR jet, to calculate the RA-RV gradient, and leads to the calculation of RVSP through the simplified Bernoulli equation [74]. However, if TR is absent, as is occasionally encountered, the assessment of RVSP becomes challenging through conventional echocardiography. It is crucial to acknowledge that RVSP estimation is subject to assumptions, such as the absence of right ventricular outflow tract obstruction and accurate assessment of RA pressure (RAP) [75]. The co-existence of RV systolic dysfunction and PH leads to the underestimation of RVSP due to two important limitations: the arbitrary set of RAP levels and the calculation of the trans-tricuspid pressure gradient, which is usually severely attenuated in severe TR. Despite the great advantage of echocardiography in easily estimating RVSP in a non-invasive way, this remains an indirect measurement of pulmonary pressures, providing a likelihood of PH and not a firm diagnosis. A detailed assessment often necessitates additional testing, particularly right heart catheterization, to provide more accurate and comprehensive insights into pulmonary hemodynamics [76].

Calculation of the RVEF and FAC is based on assumptions as well and demands meticulous manual tracings of the RV endocardial edge at both end-systole and end-diastole, and it should include the papillary muscles, trabeculations, and the moderator band [77]. Moreover, 3D echocardiography has emerged as a solution to the inherent limitations of 2D echocardiography. It surpasses these constraints in RVEF calculation, and it is considered the gold standard for global RV function evaluation. Notably, the 3D calculation of RVEF closely aligns to CMR relative measurements. It is worth mentioning that the 3D evaluation of RVEF not only strongly correlates with RV systolic function but also conclusively establishes it as an independent predictor of both cardiac mortality and major adverse cardiovascular events. The 3D evaluation of the RV may considerably contribute to this direction with potential application in patients with HHD [78]. It is crucial to know that accurate 3D echocardiography is strongly dependent on high-quality imaging, invariable heart rates, and specialized and expensive software, and demands substantial time and expertise [72].

### 3.6. Stress Echocardiography

The interplay between HFpEF and HHD is complex. Both are characterized by exertional dyspnea and effort intolerance due to elevated LV filling pressure [76,79]. HFpEF may not be clinically apparent, posing challenges to diagnosis based solely on symptoms and standard evaluations. Diastolic dysfunction is the first manifestation of this pathological process, discernible in early, mild AH even before the development of LVH. HFpEF is not uncommon among patients with HHD, but, surprisingly, hypertensive medications do not considerably alleviate symptoms or alter prognosis. Up to now, the diagnosis of HFpEF has been challenging and most recently diastolic stress echocardiography (DSTE) has been proposed in cases where diastolic dysfunction at rest is mild (grade I) [80]. On top of the resting echocardiography parameters and natriuretic peptides levels, an abnormal response during exercise testing is defined as E/e′ > 15 and an increase in TRVmax > 3.4 m/s. According to the consensus statement, DSTE may be useful to confirm or reject HFpEF diagnosis in patients at intermediate risk. However, recent questions have arisen about the reliability and technical feasibility of using this parameter during physical activity [81]. The differentiation of dyspnea among elderly patients requires a non-invasive test for HFpEF diagnosis, as patients without exercise-related diastolic dysfunction may not benefit from specific treatments [82]. Despite DSTE’s potential, the absence of diagnostic and prognostic validation, the limited expertise capacity, and the required equipment restrict its widespread use [83].

A cardiopulmonary exercise test (CPET) evaluates the cardiopulmonary system by observing how the cardiovascular and respiratory systems respond to the energy demands of muscle contraction during physical exercise. The choice of exercise modalities, such as a bicycle ergometer or treadmill, and specific protocols are personalized based on factors such as the patient’s fitness level, health, weight, and age, under the guidance of the requesting physician [84].

The combined exercise stress echocardiography (ESE) and CPET constitute a diagnostic approach aiming to unmask HFpEF in patients with AH. It is noteworthy that physical exercise has the capacity to stress cardiopulmonary homeostasis and reveal pathological hemodynamic changes that may not be apparent at rest. In addition to the aforementioned changes during DSTE, lower peak values of oxygen consumption (VO_2_) and end-tidal carbon dioxide (PetCO_2_), along with a higher ventilatory equivalent for carbon dioxide (VE/VCO_2_) slope, are independent predictors of masked HFpEF [85,86] This comprehensive diagnostic approach enables timely intervention and personalized management strategies [87,88]. Notably, CPET-ESE has recently demonstrated additional predictive value in patients with subclinical HF compared to the two techniques used separately [89]. Among its limitations, CPET-ESE is a more time-intensive and expensive procedure, requiring specialized equipment and trained personnel. Careful patient selection is critical, guided by clinical judgment. Therefore, hypertensive individuals exhibiting symptoms and signs indicative of HFpEF should initially undergo a comprehensive evaluation that encompasses standard rest echocardiography, the measurement of natriuretic peptides, and then, if it is medically deemed necessary, further exercise testing [90]. Overall, the diagnosis of HFpEF in the context of HHD may require a multimodality imaging approach to unmask symptoms and detect hemodynamic changes during exertion associated with elevated LV filling pressures [91].

## 4. Novel Echocardiographic Indices

### 4.1. Speckle Tracking of Ventricles

Myocardial strain and its sensitive index of myocardial deformation, global longitudinal strain (GLS), provides a widely accepted, accurate, repeatable evaluation of LV systolic function [92]. The sub-endocardial layer of the LV supports predominantly the longitudinal mechanics, whereas the mid-wall and sub-epicardium contribute mostly to the circumferential, rotational, and twisting motions. It is well known that AH severely suppresses LV myocardial shortening in the longitudinal direction more than in the circumferential direction [93]. Impaired myocardial deformation has long been associated with HHD [94]. Moreover, speckle tracking has the potential to differentiate various types of LVH diseases [95]. The adverse structural cardiac remodeling in HHD involves the expansion of cardiomyocytes and accumulation of fibrosis in the extracellular matrix. Speckle tracking in echocardiography has the advantage of detecting a decline in myocardial strain in patients with HHD and normal LVEF before apparent structural changes, unraveling the subtle impairment of systolic function [96]. A recent meta-analysis of six studies reported the clinical value of GLS to detect subclinical cardiac damage in patients with masked AH [97]. However, the myocardial strain is load dependent. Hence, an increase in afterload may lead to a decreased GLS, thereby attenuating the accuracy of research results [98]. A growing number of studies suggests that speckle tracking performs better than LVEF, since it may uncover subtle LV systolic dysfunction in hypertensive patients, before LVEF starts declining [99]. A compensatory modality using myocardial strain with a limited influence of blood pressure on its results is the myocardial work index [100]. This is a recently proposed, non-invasive index, based on the speckle tracking technique, to assess myocardial deformation incorporating the left ventricular pressure. Growing evidence supports the potentiality of the myocardial work index to assist the diagnostic algorithm for HHD [101,102]. The usage of specialized software is a prerequisite for the off-line analysis of myocardial work index [103]. Up to now, limited data from small studies have reported the advantages of the myocardial work index over classical echocardiographic parameters to provide a better understanding of the LV response to high BP [104]. Therefore, the combined assessment of myocardial deformation with hemodynamics could reflect all pathophysiological changes observed in HHD [105]. On the other hand, the impact of anti-hypertensive medications on this novel marker has not been fully investigated [106]. The prognostic value of alterations in myocardial work index is still unknown and requires meticulous investigation.

In addition to the diagnostic value, the contribution of GLS to prognosis has been proved in a wide spectrum of cardiomyopathies; however, its application in the risk stratification of patients with HHD has not been validated [107]. Future studies will assess the efficacy of speckle tracking to predict cardiac organ damage and determine the anti-hypertensive regimen^16^. The introduction of 3D speckle tracking echocardiography may further increase the sensitivity of echocardiography to detect regional alterations of longitudinal strain and area strain. With optimal echocardiographic views, 3D speckle tracking may assist in better quantifying the degree of myocardial damage [108]. It is a promising technique that requires further investigation.

Most recently, there is a growing body of evidence about the clinical utility of the speckle tracking of the RV [109]. It undergoes remodeling in many cardiac conditions, among them HHD [24]. However, the detection and quantification of structural and functional changes in RV is difficult, while echocardiographic images are commonly not optimal for further analysis. CMR is more advantageous for assessing the impact of hypertension on RV [110]. Under this scope, RV speckle tracking could be an alternative to obtain a better assessment of RV deformation and dysfunction in HHD with good correlation with CMR findings. Nevertheless, its clinical use remains limited, since the acquisition of images is challenging, and the prognostic value remains to be proved in large-scale studies [111].

### 4.2. Speckle Tracking of Left Atrium

**Figure 3:**
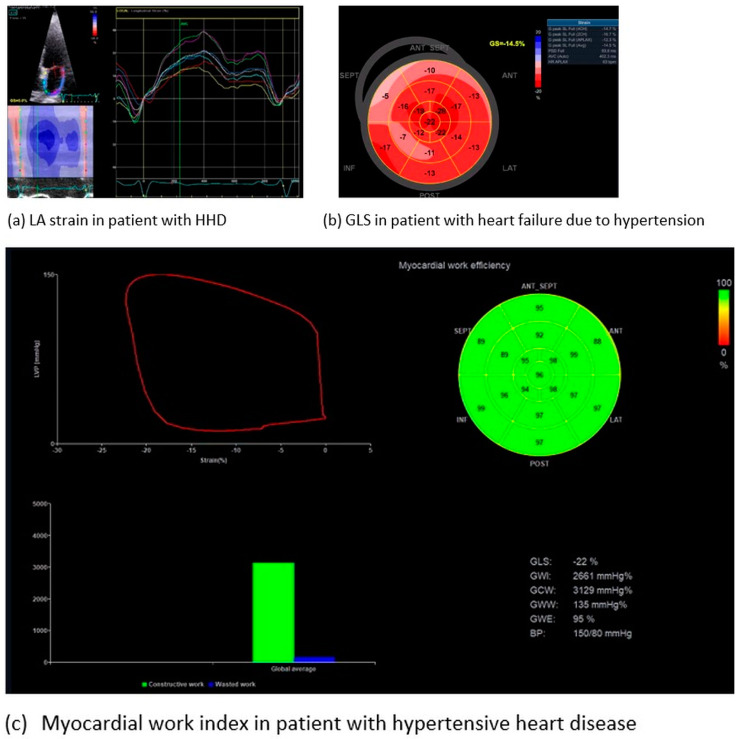
Representative images of speckle tracking in patients with hypertensive heart disease.

Most recently, researchers have hypothesized LA dysfunction before structural changes become evident, for example based on LA dilatation [112]. All three phases of LA function (reservoir, conduit, and booster pump) functions have been found to be more significantly impaired in AH patients than in healthy controls [113]. Classical (E/E′, LA size) and novel indices (reservoir strain, LASr, conduit strain, and contraction strain) of LA myocardial deformation have been associated with AH [114]. Notably, LA reservoir and booster pump strain seem to correlate with GLS in hypertensive patients. Changes in LA deformation are more sensitive than other indices, since acute blood pressure lowering in patients with hypertensive urgency promptly improves LA strain [115]. Without doubt, alterations in LA mechanics emerge early in the course of HHD development [116]. Up to now, the evidence of the diagnostic power and, to a lesser extent, of the prognostic value of LA strain in HHD has not been robust [117]. More studies are required to verify the cut-off values of LA strain. In all cases, good quality views of the LA are a prerequisite for speckle tracking implementation in order to achieve accurate and repeatable measurements (Figure 3).

Recently, both TTE and CMR have emerged stand indispensable tools for cardiac evaluation, each presenting distinctive advantages. Echocardiography, with its real-time imaging capabilities and widespread accessibility, emerges as a dynamic and cost-effective option, capable of assessing valve function and chamber dimensions. Its bedside utility and portability enhance its versatility in various clinical scenarios. Conversely, CMR, renowned for superior spatial resolution and tissue characterization, offers unparalleled insights into cardiac anatomy and function. Despite the higher costs and specialized requirements associated with CMR, its ability to provide detailed information about myocardial tissue texture and views of high imaging quality make it a valuable complement to echocardiography. The selection between these modalities hinges on clinical nuances, specific diagnostic needs, and available resources, often prompting a synergistic integration for a comprehensive cardiac assessment. The technological advances in echocardiography open new roads in its clinical application and future research will clarify its diagnostic and prognostic power.

### 4.3. Therapeutic Implications

The clinical application of echocardiography cannot only be directed towards diagnostic or prognostic purposes but can be extended to follow up hypertensive patients. Experimental data have strongly supported the favorable effects of anti-hypertensive medications on LVH [118]. Most importantly, large clinical studies have reported the association between anti-hypertensive medications, like irbesartan, and LVH and LV morphological changes [119]. The lack of LVH regression after the initiation of anti-hypertensive medications may be attributed to older age, inadequate BP control, obesity, kidney failure and longer duration of AH with delayed commitment of treatment [120]. Regarding the rest of the clinical echocardiographic parameters, the existing data are limited. Anti-hypertensive medications may also reduce LA volume [121]. Such an effect could be of clinical importance, since it may improve prognosis in non-ischemic cardiomyopathies [122]. Speckle tracking seems to provide an objective index of beneficial alterations in mechanics and geometry of the LV at the early stage of anti-hypertensive regimen [123]. Without doubt, more studies are required to demonstrate the favorable effects of anti-hypertensive medications on echocardiographic parameters in the long-term and to link those effects to clinical outcomes.

### 4.4. Gaps in Evidence and Future Perspectives

A global, widely accepted definition of non-ischemic HHD is pending. The diagnostic role of echocardiography as a first-line modality is unambiguous. However, it remains to be proved which of the proposed echocardiographic indices will cluster to set a definite diagnosis and prognosis of HHD. Significant challenges and obstacles should be addressed by echocardiographers in clinical practice to make echocardiography the gold standard technique. The novel echocardiographic parameters may assist in this direction, but they require validation. In addition to diagnostic accuracy, the relationship of echocardiography with HHD patients’ prognosis can be a new research target of pending trials, which will definitely alter the management of HHD.

## 5. Conclusions

HHD as a consequence of high-pressure overload on the heart has been increasingly recognized, constituting an emerging cardiac disease. It presumably consists of a clinical entity with unfavorable prognosis beyond CAD or kidney function impairment. Echocardiography is a first-line modality for HHD diagnosis, clustering a number of parameters of cardiac morphology and function in patients with persistent AH. The absence of a single, gold-standard diagnostic parameter has compromised all estimations of the incidence, evolution, and prognosis of HHD. The addition of novel indices, like speckle tracking of the left ventricle and left atrium, 3D volume evaluation, and myocardial work to classical echocardiographic parameters may provide more accurate and reproducible diagnostic and prognostic data in those patients. As of now, their use is still underappreciated, but they have the potential to objectively define HHD and estimate its prognosis.

## Figures and Tables

**Table 1:** Classical and novel echocardiographic indices of hypertensive heart disease.

Variable	Indices	Challenges	Indices with Prognostic Value
LVH	(1) IVSd (2) RWT (3) LVMI (4) LVEDD	Moderator band Sites of IVS measurements Wall thickness at the papillary muscles BSA or height indexing	IVS LVMI LVH pattern
LV systolic function	(1) LVEF (2) FS	Regional WMAs Geometrical assumptions Foreshortened views Endocardial delineation	LVEF
LA	(1) LA dimension (2) LAVI (3) LAVmin	Suboptimal or angled views Poor endocardial definition Foreshortening	LA dimension LAVI LAVmin
Diastolic dysfunction	(3) MV inflow pattern (4) Septal E′, lateral E′ (5) Average E/e′ (6) Pulmonary venous flow pattern	Alignment of the ultrasound beam Limited mitral annulus movement Pulmonary vein flow infrequently obtainable	E/A > 2 E/E′ ratio Pulmonary venous S/D ratio
RV systolic function	(7) TAPSE, (8) Tricupid S′ (9) RVEF or FAC (10) PASP	Variations in RV load Angle of ultrasound beam Longitudinal function of basal RV free wall Absent TR Severe TR Manual tracings of the RV endocardial edge Trans-tricuspid pressure gradient	TAPSE RVEF PASP
Diastolic stress echo	(1) E/e′ (2) TRVmax	TR detection Validation for HFpEF diagnosis	Stress E/E′ (?) Stress TRVmax (?)
Speckle tracking LV	(1) GLS (2) Myocardial work index	GLS is load-dependent Unknown cut-off values of myocardial work index	GLS
Speckle tracking LA	(1) LASr (2) Contraction strain	Optimal views of all LA walls	LASr (?)

LASr, left atrium strain reservoir; LAVmin, minimal left atrial volume; LAVI, left atrial volume index; LVEDD, left ventricular end-diastolic diameter; LVEF, left ventricular ejection fraction; LVMI, left ventricular mass index; MV, mitral valve; PASP, pulmonary artery systolic pressure; PSAX, parasternal short-axis; RV, right ventricle; RVEF, right ventricular ejection fraction; RWT, relative wall thickness; S′, systolic velocity of the tissue doppler of the tricuspid valve; TAPSE, tricuspid annular plane systolic excursion; TR, tricuspid regurgitation; TRVmax, tricuspid regurgitant maximum velocity; WMAs, wall motion abnormalities.
